# A novel, unbiased approach to evaluating subsequent search misses in dual target visual search

**DOI:** 10.3758/s13414-020-02085-0

**Published:** 2020-07-08

**Authors:** Mark W. Becker, Kaitlyn Anderson, Jan W. Brascamp

**Affiliations:** Department of Psychology, Michigan State University, 316 Physic Rd., East Lansing, MI 48823 USA

**Keywords:** Visual search, Signal detection theory, Statistical inference

## Abstract

Research in radiology and visual cognition suggest that finding one target during visual search may result in increased misses for a second target, an effect known as subsequent search misses (SSM). Here, we demonstrate that the common method of calculating second-target detection performance is biased and could produce spurious SSM effects. We describe the source of that bias and document factors that influence its magnitude. We use a modification of signal-detection theory to develop a novel, unbiased method of calculating the expected value for dual-target performance under the null hypothesis. We then apply our novel method to two of our data sets that showed modest SSM effects when calculated in the traditional manner. Our correction reduced the effect size to the point that there was no longer a significant SSM effect. We then applied our method to a published data set that had a larger effect size when calculated using the traditional calculation as well as when using an alternative calculation that was recently proposed to account for biases in the traditional method. We find that both the traditional method and the recently proposed alternative substantially overestimate the magnitude of the SSM effect in these data, but a significant SSM effect persisted even with our calculation. We recommend that future SSM studies use our method to ensure accurate effect-size estimates, and suggest that the method be applied to reanalyze published results, particularly those with small effect sizes, to rule out the possibility that they were spurious.

More than 50 years ago, radiologists first raised a concern that displays with multiple targets may result in more misses (Tuddenham, 1962). Specifically, they were concerned that the act of finding one target may reduce the likelihood of finding a second target (Berbaum, Franken, Caldwell, & Schartz, 2010; Berbaum et al., 1994; Berbaum et al., 1996). Initial evidence supported this concern, and the effect was coined “satisfaction of search.” That moniker suggested a mechanism—namely, that once a target had been found, searchers would be somewhat “satisfied” with their performance and may not search as diligently for a second target.

Although the research of this phenomenon began in radiology, visual cognition researchers have more recently started to investigate the mechanisms responsible for this decrement in second-target detection. One conclusion of that work is that there is little empirical evidence to suggest that the observed deficits in second-target detection can be attributed to less diligent searching for the second target as suggested by the satisfaction account (Berbaum et al., 1991; Cain, Adamo, & Mitroff, 2013; but see Adamo, Cain, & Mitroff, 2018). As a result, a number of researchers have argued that the mechanism-agnostic term “subsequent search misses” (SSM) is a more appropriate description of the effect (Adamo, Cain, & Mitroff, 2013; Cain et al., 2013).

Investigations into this SSM phenomenon have addressed numerous additional questions to better understand the phenomenon and its causes. This research includes investigations into whether finding one target depletes working memory (Cain & Mitroff, 2013) or causes that target’s template to become highly active in working memory (Adamo, Nah, Collegio, Scotti, & Shomstein, 2018), investigating how expertise influences the effect (Biggs & Mitroff, 2014; Cain, Biggs, Darling, & Mitroff, 2014), investigating how the effect may interact with target prevalence (Godwin et al., 2010), how the salience difference between the two targets may influence the effect (Sall & Feng, 2016), and how the effect may depend on anxiety (Cain, Dunsmoor, LaBar, & Mitroff, 2011, 2017).

Throughout these investigations, one vexing issue has been how to appropriately evaluate the presence and magnitude of the SSM effect. That is, what are the appropriate measures of single-target performance and dual-target performance to be entered into a comparison (Biggs, 2017)? To illustrate this problem, consider a scenario where you are looking for both scissors (Target A) and some tape (Target B). As a natural starting place, you open your junk drawer and start searching through the clutter for both items. The primary question of interest in the SSM literature is whether finding the tape reduces your subsequent ability to find the scissors. In the lab, a typical experiment designed to investigate this issue would have target-absent trials, single-target trials (with either Target A alone or Target B alone), and dual-target trials (with both Target A and Target B). Given the primary question of interest, the critical comparison would be between detection rates when Target A appeared alone and detection rates for Target A *following the detection of Target B* in dual-target trials. However, it would be inappropriate to directly compare the total number of Target A detections in these two cases, as the latter’s contingency of having found Target B prior to finding Target A adds a second requirement. The conjunction of two events must be less than (or equal to, in the limiting case) the likelihood of either event occurring alone (Tversky & Kahneman, 1974).

To address this concern, Biggs (2017) considered a number of methods for calculating a corrected value of second-target detection performance to allow a direct comparison to detection performance in the single-target condition. While a number of calculations were considered, all had the goal of removing the impact of the contingency from the dual-task calculation, and all did this by calculating dual-task accuracy considering only those trials were Target B was detected first. In other words, these methods compared search accuracy on Single-Target A trials to the contingent probability P(A I B) for dual-target trials. The following is the most widely adopted formula for calculating this contingent probability:

1 $$ \mathrm{F}1:\frac{\mathrm{trials}\ \mathrm{with}\ \mathrm{B}\ \mathrm{then}\ \mathrm{A}\ \mathrm{detected}}{\mathrm{all}\ \mathrm{trials}\ \mathrm{wit}h\ \mathrm{B}\ \mathrm{detected}\ \mathrm{first}\ } $$

On its surface, this calculation seems well founded; considering only those trials where Target B has already been found eliminates the contingency of having to find B before A. Given its intuitive appeal, this approach (or a variation on this approach) has been widely adopted.

However, Adamo, Cox, Kravitz, and Mitroff (2019) recently pointed out that that approach introduces a systematic bias, leading to a consistent underestimation of second-target performance. As a result, the approach can lead to an overestimation of the size of the SSM effect. At a conceptual level, the main problem with the traditional method (Biggs, 2017) of calculating second-target search performance for a Target A is that it considers only trials where A is found *following detection of B*. Critically, the calculation therefore eliminates trials where Target A is detected prior to Target B. Arguably, these eliminated trials would be, on average, cases where Target A is “easy” to find. (i.e., is found rapidly). By contrast, the calculation of Single-Target A performance would include all cases, both easy and hard. This difference in inclusion criteria leads the calculation to be systematically biased against the second target condition.

One way of addressing this issue is by trying to limit the calculation of Single-Target A performance to difficult cases in a way that is matched to the calculation of second-target performance. This is the approach Adamo et al. (2019) took in an attempt to circumvent the bias in the original method. Their experiment included matched sets of three trials that all shared the same target and distractor layout; one contained both targets, one contained only Target A with Target B replaced by a distractor, and one contained only Target B with Target A replaced by a distractor. All trials were randomly interleaved. Motivated by the fact that the second-target performance calculation includes only a subset of, mostly difficult, two-target trials, this design allowed Adamo and colleagues to use only the *corresponding* single-target trials in their Single-Target A performance calculation, thereby attempting to match difficulty across both calculations. This would be an excellent approach if a large majority of the variability in search difficulty could be accounted for by display layout. However, it is possible that other factors also substantially contribute to difficulty for a given target on a given trial (e.g., trial history, momentary shifts in vigilance, internally generated changes in activation of one of the search templates, etcetera). This means that an approach centered on equating search array layouts may be rooted in an incomplete characterization of the underlying factors that determine search difficulty. As a result, such an approach may suffer from the same issue as the traditional approach and result in SSM estimates that are biased in the same way. We will detail below that this is, indeed, the case with the approach proposed by Adamo et al. (2019).

In what follows, we will present a method that, we argue, provides an unbiased estimate of SSM magnitude. Two key features of the method are, first, that it takes care of the concern of biased trial selection with regard to search difficulty; and, second, that it does so without committing to any particular factors that might determine this difficulty. Instead, and importantly, the method treats the process of searching for a target as fundamentally stochastic in the sense that search difficulty might vary from trial to trial due to various factors that cannot be fully controlled, and it incorporates this stochasticity in its computations rather than relying on attempts to control those factors.

After describing the logic behind our method, we will examine the impact of the above-described bias on SSM estimates by applying both the traditional approach and our novel approach to empirical data collected in two SSM experiments in our lab. Because those particular data are not suitable for applying the method proposed by Adamo et al. (2019), we will also apply our method to a published set of Adamo et al.’s (2019) data to directly observe how our approach compares to those authors’ approach to addressing the systematic bias in the traditional method.

To preview, we find that our method results in substantially smaller SSM effect estimates than the traditional method (Biggs, 2017) as well as the recently proposed alternative (Adamo et al., 2019), and that apparent SSM effects in our own data sets, but not in the published data set, disappear when using our method. We also demonstrate that display layout does not account for much variability in search difficulty in the published data set, which explains why our method leads to smaller SSM estimates for those data than Adamo et al.’s (2019) method centered on controlling display layout. We conclude with a set of recommendations for researchers investigating SSM effects to follow, in order to avoid biased calculations that can overestimate SSM effect sizes, and could even produce spurious SSM effects.

## A different approach to avoiding SSM estimation bias

Our approach is different from existing approaches in that it is not aimed at directly comparing Single-Target A performance to a measure of second-target performance and, consequently, it does not involve any attempt to match those two performance measures in terms of the search difficulty of the underlying targets. Instead, our approach treats search difficulty for a given kind of target as inherently a stochastic variable that has some distribution—that is, that has some across-trial variability that we do not attempt to control (using display layout or otherwise). Our method, then, is aimed at including estimates of that variability, obtained from the empirical data, in our prediction of what the quantity proposed by Biggs (2017; our Formula 1) would be under the null hypothesis that no SSM effect exists. In other words, there is no attempt to arrive at an unbiased alternative to Biggs’s (2017) variable, nor to arrive at an equivalently biased measure of Single-Target A performance to use for comparison (as in Adamo et al., 2019). Instead, we predict Biggs’s variable, including whatever bias it may have, under the assumption that an SSM effect is absent, and any deviation between this prediction and the actual variable counts as evidence for an SSM effect.

**Fig. 1:**
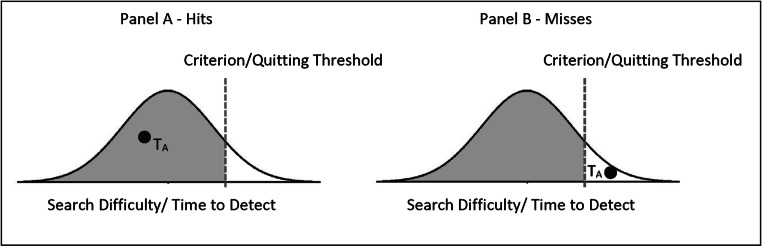
To adopt a signal detection theory (SDT) framework to visual search responses, we posit that search difficulty, which can be thought of as the time required to find a target is normally distributed across trials. Like traditional SDT, there is a decision criterion, which in the visual search literature is analogous to a quitting threshold. If the target for a given trial is drawn from the part of the distribution of target difficulties to the left of the quitting threshold (**a,** shaded area), the target will be detected prior to hitting the quitting threshold. If the target is drawn from the part of the distribution to the right of the quitting threshold (**b,** unshaded area), the trial will be terminated before detecting the target and there will be a miss

To introduce our approach we will now revisit the source of bias in the traditional method using a framework inspired by signal-detection theory (SDT). To adopt SDT to visual-search responses, we assume that search difficulty, or the time required to detect the target, for Target A is normally distributed across trials (the actual shape of this distribution is not critical). Further, there is a decision criterion, which in the domain of visual search can also be considered a quitting threshold. If the target for a given trial is easy to detect (see Fig. 1a), the target will be successfully detected prior to reaching the quitting threshold and the result will be a hit. By contrast, if the target for a given trial is sufficiently difficult to detect (see Fig. 1b), the quitting threshold will be reached before finding the target, and the result will be a miss. Thus, the shaded area under the curve represents the hit rate, and the nonshaded area represents the miss rate.

**Fig. 2:**
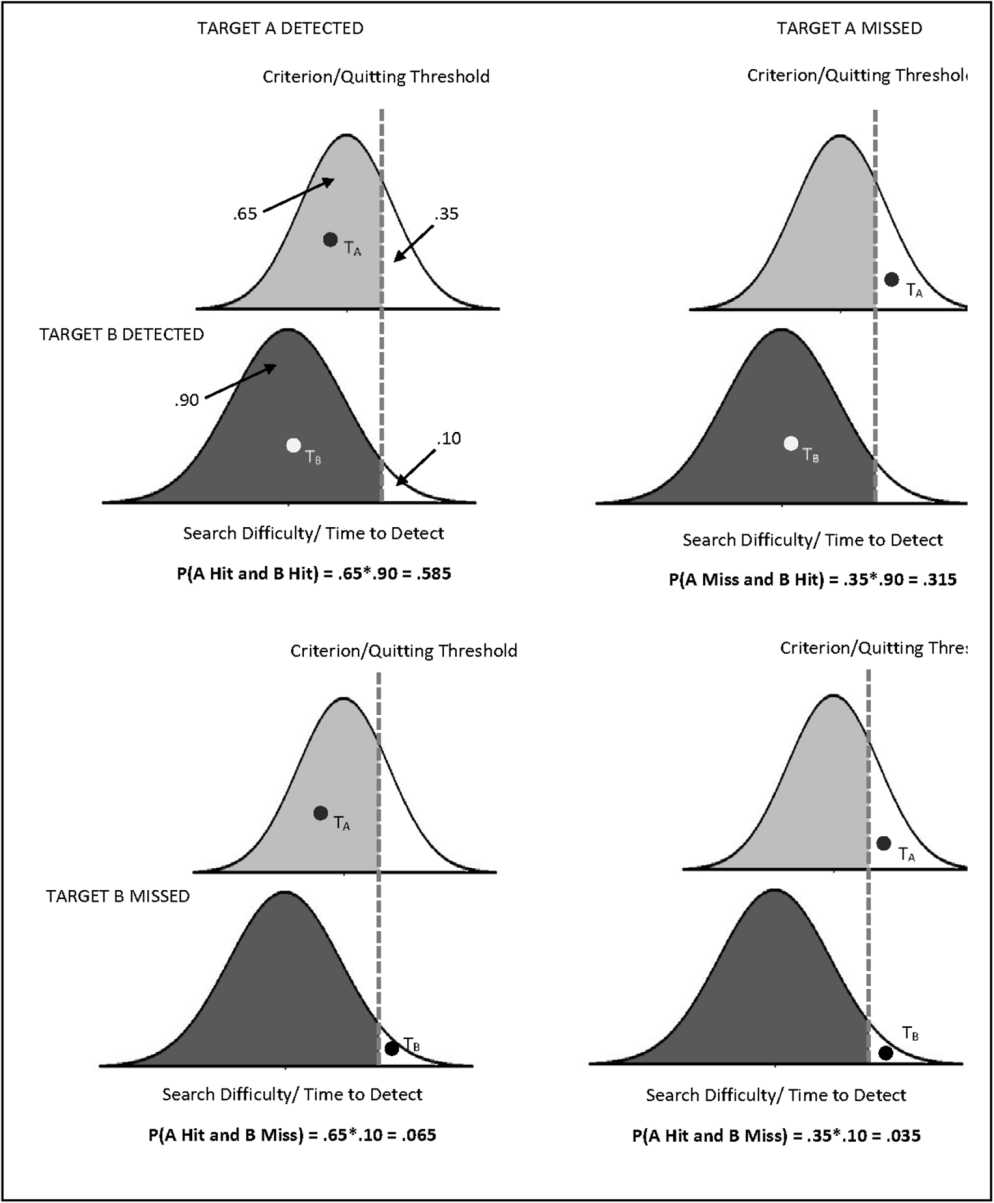
The figure illustrates how one can adopt a SDT approach to a dual-target search scenario. The distributions with light-gray and dark-gray shading correspond to the distribution of Target A and Target B difficulties, respectively. The four quandrants of the figure represent the 2 × 2 factorial combination of the response of detecting or missing Target B (rows) and detecting or missing Target A (columns). In the example we assume a Single-Target A hit rate of 65% and a Single-Target B hit rate of 90%. While this signal-detection model provides estimates of likelihood of the four possilble types of response, it is worth noting that the upper-right quadrant (detect both targets) is composed of a portion of trials in which A is detected before B and in which B is detected before A. Note that the distribution for Target A and B can have different means standard deviations, but the quitting threshold is the same for both targets because it is set for the entire trial

To expand this logic to trials with two targets gets a bit more difficult. For illustrative purposes, assume a scenario where the single-target hit rates are .65 for Target A and .9 for Target B. The application of SDT and basic rules of probability allow us to use these single-target detection rates to estimate the likelihood of four possible dual-target trial outcomes. As illustrated in Fig. 2, these are finding both A and B (top left); finding only A (bottom left); finding only B (top right); and finding neither (bottom right). Note that these predictions are under the null hypothesis that the detection of one target has no impact on the detection rate of the other.

However, none of the four calculated probabilities illustrated in Fig. 2 is a direct prediction of the probability of detecting A *after detecting B*, because none consider the order of target detection. Rather, the calculated probability of finding both targets in a trial (the upper left panel of the figure) is a composite of two types of trials—those where A is detected after detecting B (the data of interest) and those where A is detected before detecting B. As such, this calculated probability of detecting both A and B sets the upper limit of the expected probability for detecting A after detecting B. The proportion of A following B trials will fall below this upper limit by an amount that corresponds to the proportion of trials in which Target A is detected before Target B. For instance, assume that B was the first target detected in 80% of the trials where both targets were detected, and A was detected first in the other 20%. Then, given that the expected proportion of trials where both targets were detected was .585 (from upper-left panel of Fig. 2), only 80% of them, or .468, would be expected to be trials where A was detected following B. In short, and this is the key observation here, with knowledge of the proportion of dual detections that follow the B then A ordering, we can derive an expected value for the rate of B then A detection under the null hypothesis that finding B does not impact the ability to find A.

Before turning to an examination of how we can predict the proportion of dual-target trials with a specific order of detection, we use the example we have developed to empirically demonstrate the bias inherent in the traditional calculation of second-target detection rates. To refresh, that calculation considers only trials where Target B is detected in the calculation. As such, the formula (see Formula 1, above) can be rewritten as follows:

2 $$ \mathrm{F}2:\kern0.5em \frac{\mathrm{trials}\ \mathrm{with}\ \mathrm{B}\ \mathrm{then}\ \mathrm{A}\ \mathrm{detect}\mathrm{ed}}{\mathrm{trials}\ \mathrm{with}\ \mathrm{B}\ \mathrm{then}\ \mathrm{A}\ \mathrm{detect}\mathrm{ed}+\mathrm{trials}\ \mathrm{with}\ \mathrm{only}\ \mathrm{B}\ \mathrm{detect} ed} $$

For our example that we just calculated, the expected value for “trials with B then A detected” would be .468 (assuming a B then A ordering of detection in 80% of the trials where both targets were detected). Thus, inserting the values from our probability calculations, the expected value of this traditional calculation would be .468/(.468 + .315) = .59. The traditional method would then directly compare this calculated value with the single-target detection rate. Note, however, that this .59 is below the .65 single-target detection rate for Target A that was used to generate our expected values in our example. Thus, this apparent SSM effect of 6% is due to bias in the calculation rather to than any actual effect that finding Target B had on finding Target A.

**Fig. 3:**
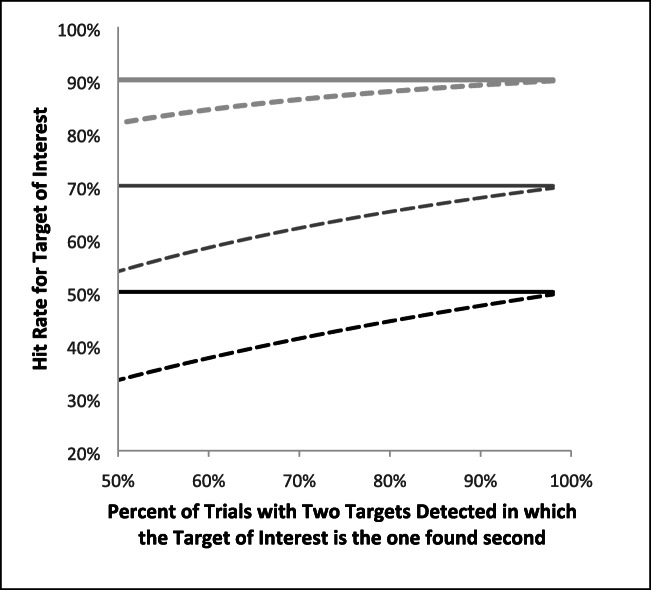
Solid lines represent three different levels of single-target detection accuracy. Broken lines are the corresponding expected values for second-target accuracy for the target as computed by the traditional calculation. The traditional method is accurate when the target of interest is found second in all dual-target trials where two targets are detected. However, bias is introduced and increases as the percentage of trials where the target of interest is found second decreases, and this bias is exaccerbated as the single-target accuracy for the target of interest decreases

While the above example is a single example, in Fig. 3 we calculated the bias in the traditional measure across a variety of Single-Target A detection rates (*y* values of solid lines) and a variety of B then A rates (finding B before A in dual-detection trials; *x*-axis). As can be seen from Fig. 3, broken lines, the traditional calculation is accurate when B is found before A on all dual-target trials, but systematic bias increases as the number of trials on which A is found prior to B increases. Intuitively, this pattern makes sense, given that the main problem with the traditional calculation is that it throws out successful target detections when the target of interest (A, in this example) is found first. Figure 3 also demonstrates that the magnitude of this bias is dependent on the single-target hit rate for Target A; as the hit rate for Target A decreases, the magnitude of the effect increases.^1^

In short, this exercise of calculating the second-target detection rates using expected values generated under the null hypothesis and the traditional method of calculation provides a couple of key insights. First, it confirms the inherent bias identified by Adamo et al. (2019) in the measure of second-target performance that has been used, which renders the measure unsuited for direct comparison to single-target performance. This bias could create apparent SSM effects when none are present, or artificially increase the magnitude of the effects when they are present. Second, the magnitude of the bias depends on two critical factors: the single-target hit rate for the target of interest (Target A in our example) and the proportion of trials in which the target of interest is found before the other target when both are detected, a quantity we term the reversal rate. Finally, and critically for our present purposes, if these two factors are known, one can use that knowledge to make estimates of the variable proposed by Biggs (2017; i.e., the proportion of trials detecting Target A following Target B) under the null hypothesis that no SSM effect exists—against which observed dual-task performance can be evaluated. The following section describes this estimation process in detail.

## Making unbiased dual-task performance predictions

One way to derive a prediction of second target detection rate under the null hypothesis is to use the single-target data to calculate the probability of finding both Target A and Target B (regardless of order) in dual-target trials, and then to use the observed reversal rate (% of dual detection trials that do not occur in the order of interest) to scale this probability. This is fairly similar to the example we performed above, and requires three pieces of information: the single-target hit rate for the target of interest [P(A)], the single-target hit rate for Target B [P(B)], and the proportion of trials, out of only those two-target trials where both targets are detected, in which Target A is found after Target B [P(A after B)]. Provided these three measures, the expected proportion of all two-target trials on which A is found after B, under the null hypothesis, is given by the following formula:

3 $$ \mathrm{F}3:\kern0.5em \mathrm{P}\left(\mathrm{A}\right)\ast \mathrm{P}\left(\mathrm{B}\right)\ast \mathrm{P}\left(\mathrm{A}\ \mathrm{after}\ \mathrm{B}\right) $$

Of course, to directly compare this value to the traditional calculation, one would have to put this value over the denominator of all trials in which Target B was found first, so the formula to calculate a value that can be directly compared with the traditional calculation would be:

4 $$ \mathrm{F}4:\kern0.5em \frac{\mathrm{P}\left(\mathrm{A}\right)\ast \mathrm{P}\left(\mathrm{B}\right)\ast \mathrm{P}\left(\mathrm{A}\ \mathrm{after}\ \mathrm{B}\right)}{\mathrm{P}\left(\mathrm{A}\right)\ast \mathrm{P}\left(\mathrm{B}\right)\ast \mathrm{P}\left(\mathrm{A}\ \mathrm{after}\ \mathrm{B}\right)+P(B)\ast P\left( not\ A\right)} $$

Most SSM experiments have single-target conditions, so the P(A) and P(B) can be calculated directly from the single-target trials. The P(A after B) term can also be calculated directly from the dual-target trials. Thus, it would be relatively easy to perform these calculations based on the data typically collected in an SSM experiment.

Note that the reversal rate is a quantity that is directly related to the across-trial variability in search difficulty for the Targets A and B: It depends on the degree of overlap between search difficulty distributions for the two targets. So, by incorporating the empirically observed reversal rate into our computation, we are taking into account knowledge of this variability without trying to control it. However, we will also apply a second method in addition to the one just described, based on the following consideration: it is somewhat odd to use the dual-target data themselves to determine the reversal rate term P(A after B), and then use that term to predict dual-target performance. The possibility arises that this prediction is not independent from the data to which the prediction is applied. In an ideal situation, one would infer the nature of across-trial variability in search difficulty and, ultimately, derive a prediction of dual-target performance, entirely on the basis of single-target data.

**Fig. 4:**
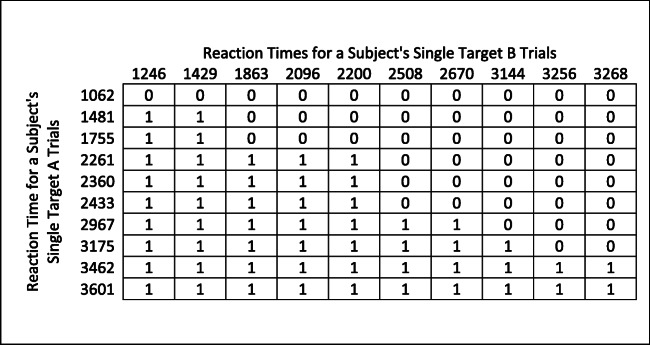
How to use single-target RTs to derive and estimate of the expected probability of dual-target detection trials in which the order would be to detect Target B before Target A. For any cell where the Target A reaction time was greater than the Target B reaction time, we would assign a 1; for all other cells we assign a zero. The average of this matrix provides an estimate, based on single-target trials, of the proportion of dual-target detection trials in which Target A would be found after finding B

This turns out to be possible, provided one collects reaction time data for the single-target trials. In particular, given that single-target displays and dual-target displays in a given experiment are made up of the same kinds of targets and distractors, one can reasonably use the single-target reaction-time distributions for Target A and Target B to estimate the probability that a given Target B would be detected before a given Target A in a dual-target trial. One way of doing this is to simulate a set of hypothetical dual-target trials, each corresponding to a combination of one actually observed Single-Target A trial and one actually observed Single-Target B trial. In this case, for a given observer one would make a matrix of all the successful Single-Target A detection reaction times by all successful Single-Target B detection reactions times (see Fig. 4). If a 1 was entered in every cell where the RT for A was greater than the RT for B (i.e., a simulated trial where Target B would be found first), and a zero elsewhere (i.e., a simulated trial where Target A would be found first), the average of the matrix would be an estimate, based solely on the single-target data, of the proportion, out of those dual-target trials in which both A and B are found, in which A should be detected after B. This proportion then replaces the “P(A after B)” term in Formulas 3 and 4, above. In this way, the prediction of dual-target performance can be calculated relying completely on the single-target data.

## An empirical test

Having derived the above suggestions for analyzing SSM data, we applied both of our proposed methods (as well as the traditional approach) to empirical data from two SSM experiments that we had run in the lab. The two experiments were originally designed to investigate whether the SSM resulted because detection of one target made that target active in working memory, thereby biasing subsequent attention toward features of that target, and away from features of a different target (Gorbunova, 2017). However, we use them here simply to evaluate how our unbiased method influences SSM analyses and interpretations. Importantly, these data showed significant SSM effects when analyzed with the traditional method, thereby providing an opportunity to investigate the extent to which that significant effect may be reduced or eliminated by analyzing the data with the unbiased methods we derived here.

## Methods

### Participants

Subjects were recruited from Michigan State’s undergraduate research pool; subjects were granted course credit or extra credit for their involvement in the experiment. The subjects’ age ranged from 18 to 35 years, and they had normal or corrected-to-normal vision. For the analyses we perform here, data from a subject was excluded from analysis if the subject’s accuracy was 2.5 standard deviation away from mean. This criterion resulted in useable data from a total of 59 subjects (26 in Experiment 1 and 33 in Experiment 2).

### Apparatus and stimuli

The experiment was coded using Experiment Builder software (SR Research, Ottawa, ON, Canada) for the EyeLink 1000 (SR Research), and presented on a 27-inch monitor with 1,024 × 768 resolution. A head rest placed 66 cm away from the computer screen stabilize the head, while the participant’s right eye was tracked. The experiment began with a calibration and validation procedure for the eye tracker.

**Fig. 5:**
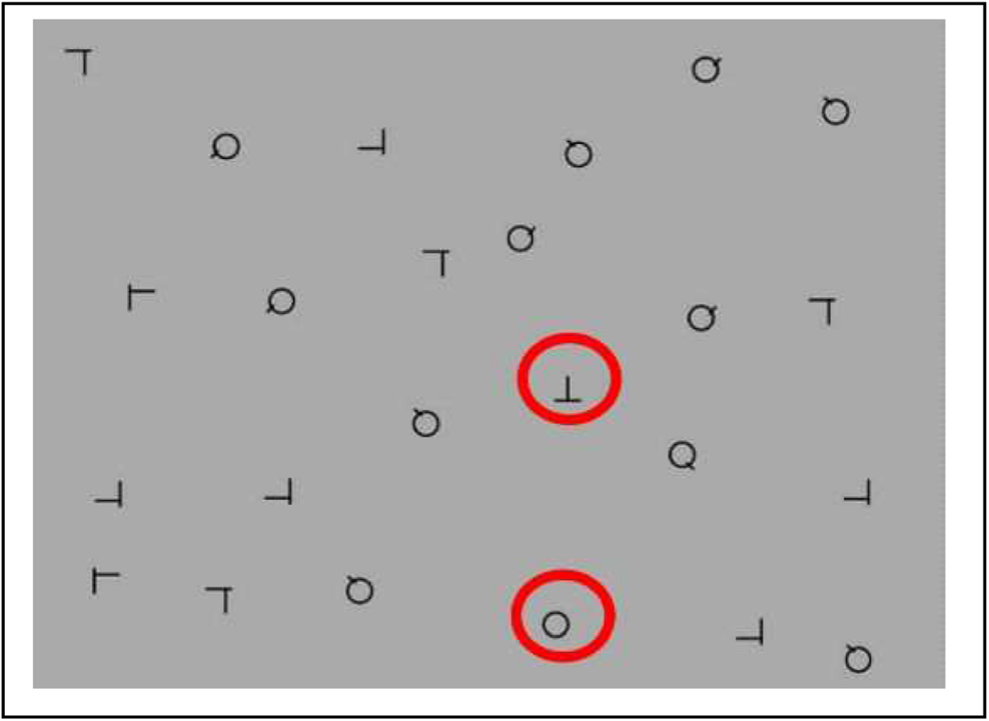
An example of a dual-target search array with two different targets (T and O). The targets here have been circled for illustrative purposes, but they were not in the actual experiment

Each search array consisted of 24 stimuli (each ~.92° × .82°of visual angle) that were distributed in a 6 × 4 grid, with each object’s location randomly jittered within that section of the grid (see Fig. 5). Target-absent displays were composed of an equal number of *Q*s and offset *L*s which could appear in any of four rotations and which were randomly dispersed throughout the array. Targets were *O*s and rotated *T*s. There were six different types of search arrays: absent, a single *T*, single *O*, two *T*s, two *O*s, and *T* and *O* search arrays. The subjects were informed there could either be one target, two, targets or no targets present.

### Procedure

Every trial began with a central fixation point that checked the calibration of the eye tracker. Following fixation on the central fixation point, the search array appeared and remained present until subjects terminated the trial. Subjects were instructed to press one button every time they detected a *T*, a second button for every *O*, and a third button to indicate that there were no more targets (i.e., terminate the trial).

Both experiments were identical, except for the number of trials in each condition. In Experiment 1, there were three blocks of trials; the first block was a practice block consisting of 16 trials (four absent, two single *T*s, two single *O*s, two double *T*s, two double *O*s, and four *TO* trials). The next two blocks were test blocks each had 64 trials composed of 16 absent, eight single *T*s, eight single *O*s, eight double *T*s, eight double *O*s, and 16 *TO* trials that were randomly interleaved.

In Experiment 2, the practice block consisted of two search arrays of each of the six conditions (absent, sing *T*, single *O*, double *T*, double *O*, and *TO* trials). The test blocks were composed of 72 trials each, with 24 absent, 12 single *T*, 12 single *O*, eight double *T*, eight double *O*, and eight *TO* trials. For the analyses used here we only used the single-target and *TO* trials.

## Results

The main data of interest for evaluating our method of analysis were the data from the single-target conditions, and the condition in which two distinct targets appeared (*TO*). We performed identical analyses for Experiments 1 and 2. We used traditional inferential statistics, but when comparisons suggested null effects, we additionally calculated the Bayes factor. For the Bayes factor calculation, the null hypothesis was that the two conditions had the same mean, and the a priori distributions were set to be uniform distributions. When reporting Bayes factors, we report them in terms of the likelihood of the null over the alternative hypothesis. This method is more intuitive when trying to evaluate support for the null hypothesis, as the Bayes factor reported in this way indicates the how much more likely the null hypothesis is than the alternative hypothesis.

**Fig. 6:**
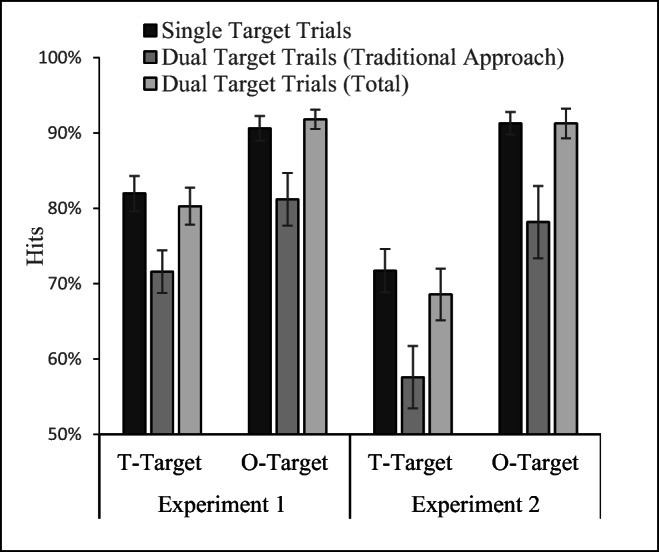
Mean single-target accuracy is compared with the observered dual-target accuracy using the Biggs (2017) adjustment of dual-target accuracy and to the total dual-target accuracy (regardless of detection order). Error bars indicate the standard error of the mean

For each experiment, we calculated each subject’s single-target hit rate and dual-target hit rate (see Fig. 6) using the traditional method of calculation suggested by Biggs (2017). Then, as a first pass to addressing the bias in this method, we also calculated the total observed rate of target detections for each target during dual-target trials, regardless of any other considerations (i.e., including trials were the other target was found second and where it was missed). After all, recall that the bias arises from eliminating trials where only the target of interest was detected and those where the target of interest was detected prior to the other target. Computing the overall hit rates for the two targets during dual-target trials (regardless of order) does not involve any such eliminations, so it is a useful, albeit coarse, first pass approach. If it is true that the presence and/or detection of another target during dual-target trials has no bearing on search for a given target (i.e., that there is no SSM effect), then these overall detection rates should match single-target detection rates. A 2 (target identity *O* or *T*) × 3 (method of calculation: single-target hit rate, traditional dual-target hit rate, or overall dual-target hit rate) repeated-measures ANOVA performed for each experiment (see Fig. 6) found significant main effects of target identity, Experiment 1, *F*(1, 25) = 14.23, *p* = .001; Experiment 2, *F*(1, 32) = 58.44, *p* < .001, with better detection of the *O* target than the T target. Critically there was also a main effect of method of calculation, Experiment 1, *F*(2, 50) = 23.51, *p* < .001; Experiment 2 *F*(2, 64) = 17.556, *p* < .001. The two factors did not interact (*F* < 1 for both experiments).

Paired *t* test were used to investigate the source of the main effect of method of calculation (see Table 1). These comparisons consistently show a significant SSM effect when comparing the single trial performance to the second-target performance calculated using the traditional method suggested by Biggs (2017). That is, if we had performed the traditional analyses on these data, we would have concluded that there were significant SSM effects. However, comparing single-target detection rates to the total-target detection rates (regardless of order of detection) in the dual-target trials, we found no significant differences. In addition, the Bayes factors for these null results correspond to substantial evidence in favor of the null hypothesis. In sum, the traditional method of evaluating the SSM effect (comparing the left bars to the middle bars in Fig. 6) would have led to the conclusion that there was a significant SSM effect in our data; however, that conclusion would not be reached when using overall detection rates on all two-target trials regardless of other considerations (see the right bars in Fig. 6). One possible reason for this difference is the bias we identified in the traditional method of calculation; if one does not eliminate hits form the dual-target trials based on order of detection, there is no evidence for reduced detections in the dual-target trials. In short, the calculation of overall dual-target detection rates, regardless of order, is a good first test to evaluate in an unbiased manner whether SSM effects are present in a data set, but for a more powerful test we turn to the two methods we derived above. In particular, we derived expected values for the traditional calculation (in the absence of any true SSM effect) based on both of our methods of prediction (i.e., using observed dual-target reversal rates and using single-target RT distributions to estimate reversal rates). These predictions were made at the level of the subject.

**Table 1:** Statistics for the paired *t* test and Bayes factor comparisons

		Single vs. dual 2nd target (traditional)	Single vs. dual total	Dual 2nd target (traditional) vs. E.V. under H0 (RT method)	Dual 2nd target (traditional) vs. E.V. under H0 (obs. reversal rate)
T Targets	Exp. 1 *df* = 25	*t =* 4.44, *p* <.001 Bayes = .006	*t =* .85, *p* = .40 Bayes = 4.68	*t =* .18, *p* = .86; Bayes = 6.51	*t =* .44, *p* =.66; Bayes = 6.02
	Exp. 2 *df* = 32	*t =* 3.20, *p =*.003 Bayes = .098	*t* = .88, *p =*.39 Bayes = 5.11	*t =* .72, *p* = .48; Bayes = 5.78	*t =* .12, *p* = .90, Bayes = 7.35
O Targets	Exp. 1 *df* = 25	*t* = 2.60, *p* = .015 Bayes = .361	*t =* .75, *p* = .46 Bayes 5.06	*t =* .50, *p* =.63; Bayes = 5.88	*t =* .07, *p* =.95; Bayes = 6.61
	Exp. 2 *df* = 32	*t* = 2.84, *p* = .008 Bayes = .221	*t =* 0, *p* = 1 Bayes 7.41	*t =* .34, *p =*.74; Bayes = 7.00	*t =* 1.02, *p* = .32; Bayes = 4.50

*Note.* Bayes factors are expressed as the ratio of H0 to H1, such that a Bayes factor of 7 indicates that the null hypothesis is 7 times more likely than the alternative hypothesis. E.V. stands for the expected value as calculated using our formulae.

**Fig. 7:**
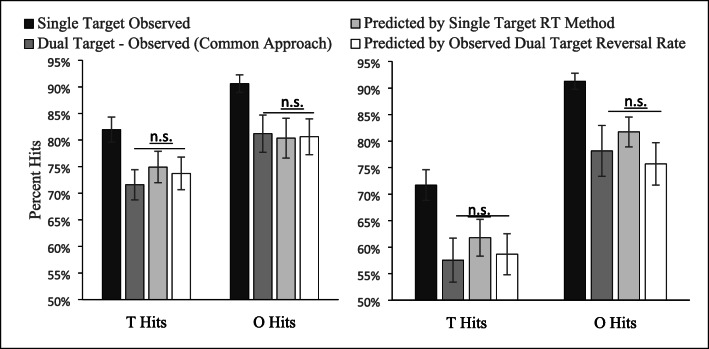
Black bars depict the single-target accuracy rates and are provided to show that the traditional method of calculating dual-target performance (dark-gray bars) is lower than that of the single-target performance. Of more interest are the two lighter bars that represent the expected values for the traditional dual-target calculation derived under the null hypothesis. As is clear, observed dual-target performance is very similar to the expected perforance under the null. The left-hand panel depicts Experiment 1 and the right-hand Experiment 2. Error bars are the standard error of the means

Figure 7 plots the observed second-target detection rates calculated using the traditional method and the expected values for these observed rates derived under the null hypothesis. If there is truly an SSM effect, then the second-target detection rates should be lower than these expected values. By contrast, if there is no SSM effect, we should find no significant difference between the second-target detection rates and the derived expected values for these detection rates. We have also replotted the single-target detection rates in the figure to depict the SSM effect that would be inferred if one followed the traditional method of comparing the second-target detection rates to the single-target detection rates. However, the real comparison of interest for our present purposes is between the observed second-target detection rates (calculated with the traditional method) and the expected values for these rates that we derived using the methods described above.

A 2 (*T* target/*O* target) × 3 (calculations: observed dual target, predicted dual target based on RT, predicted dual target based on probability) repeated-measures ANOVA was performed for each experiment. The pattern of results was identical across experiments. In each case there was a main effect of target letter, Experiment 1, *F*(1, 25) = 5.92, *p* = .022; Experiment 2, *F*(1, 32) = 27.84, *p* < .001, no main effect of type of calculation, Experiment 1, *F*(2, 50) = .157, *p* = .86; Experiment 2, *F*(2, 64) = 1.57, *p* = .22, and no interaction, Experiment 1, *F*(2, 50) = .377, *p* = .69; Experiment 2, *F*(2, 64) = .24, *p* = .79. In both experiments, the main effect of target letter resulted because the *T* target was harder to detect than the *O* target. Critically, the lack of a main effect of type of calculation suggests that there is no evidence of an SSM when the observed second-target detection rates calculated using the traditional approach are compared with expected values for these calculations derived under the null hypothesis. Even so, we provide paired *t* test for each comparison and the Bayes factors for each comparison in Table 1. Of note, none of the paired comparisons approach significance and the Bayes factors range from 4.5 to 7.35, suggesting that that null hypothesis is more than 4.5 times as likely as the alternative hypothesis. Note that all these values for the Bayes factor would conventionally be interpreted as substantial evidence for the null hypothesis (Dienes, 2014). In short, the observed second-target detection values are not significantly different from the expected values under the null hypothesis (under either method of predicting switch rates) and substantial evidence indicates that they are, in fact, the same. These findings suggest that, in our two data sets, the significant differences between the single-target and second-target detection performance measures that were found with the traditional method of comparison were completely accounted for by the bias that is inherent in the second-target calculation and, therefore, that there is no SSM effect in our data.

A final method of evaluating whether there is a true SSM effect is to parcel the dual-target trials into all five possible outcomes of dual-target trials (i.e., taking into account hit order) and compare the observed and expected percentages for each type of response. Again, we calculated these expected percentages at the subject level, and we did so by extending the two methods introduced above to predict all five possible outcomes. The first method of prediction used the RT data from the single-target conditions and nothing else to estimate the expected responses for the dual-target data under the null hypothesis. The second method calculated the expected probabilities for the dual-target data using probability theory based on single-target hit rates and the observed reversal rate in the dual-target trials.

**Fig. 8:**
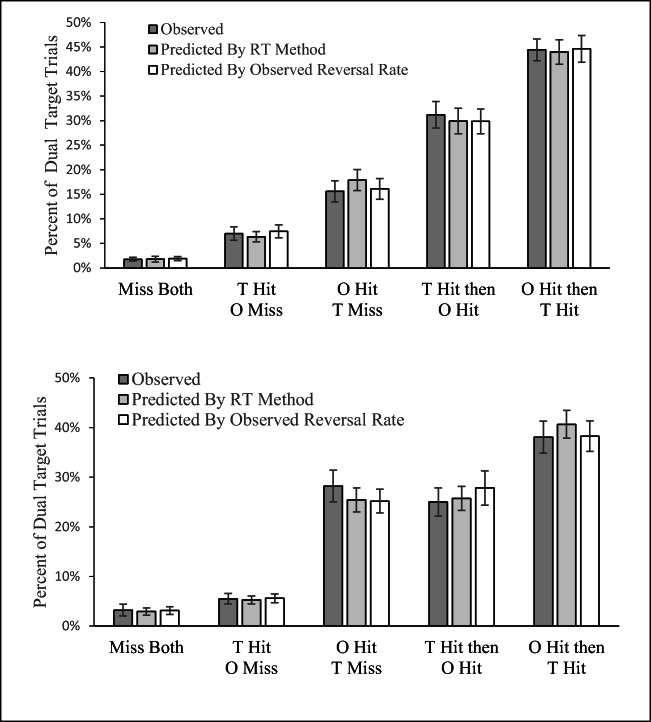
Comparison of the observed mean percentage of each type of response in dual-target trials to the predicted likelihood of each type of response under the null hypothesis for Experiments 1 (top) and 2 (bottom). The observed dual-task performance is in line with predictions under the null hypothesis, whether those predications were based on our method, or modeling based on single-target RT data, or based on our probability calculations. Error bars are the standard error of the means

Figure 8 presents the observed and predicted percentages for each type of trial outcome in both experiments. Again, the pattern of data is very consistent across experiments. As is clear from the figure, the observed data are extremely similar to the predicted data, which were made under the null hypothesis. This analysis provides additional evidence that there is no SSM effect when the expected value for dual-target performance is calculated correctly. To verify this interpretation, we ran a series of five repeated-measures ANOVAs comparing the observed proportions for each classification of response with the two predictions, for each experiment. For Experiment 1, none of the five comparisons approached significance, all *F*s(2, 50) < 1.14, all *p*s >.32. Similarly, for Experiment 2, none of five comparisons approached significance, all *F*s(2, 62) < 1, all *ns*.

In short, this approach also finds no evidence of a SMM effect. For good measure, we again calculated Bayes factors for all pairwise comparisons within each response classification. The smallest Bayes factor across all these comparisons was 3.52 for Experiment 1 and 4.50 for Experiment 2. This again translates to substantial evidence in favor of the null hypothesis that observed two-target performance is as predicted in the absence of SSM effects.

## Discussion

Across two experiments, analyzing our data using the standard method (described by Biggs, 2017) of comparing single-target detection performance to second-target detection performance showed a significant SSM effect. However, that significant SSM effect was attributable to a bias inherent in that standard method of calculation. When single-target performance was compared with dual-target performance regardless of the order of found targets, and also when observed dual-target performance was compared with unbiased estimates of the expected value for dual-target performance, there was no evidence for an SSM effect. In addition, our two methods of calculating expected values for dual-target performance were able to capture the relative proportions of all five possible dual-target outcomes quite well. Taken in total, our results indicate that the significant SSM effects for both experiments that were obtained using the standard method of calculation were spurious findings that disappeared when avoiding the biased method of calculation.

## Application to Adamo et al.’s (2019) method

Next we applied our method to data from the different saliency experiment in Adamo et al. (2019). We did this for two reasons. First, the traditional calculation of the SSM effect size in that paper was a Cohen’s *d* of 1.63. This is substantially larger than the largest effect size in our two experiments using the traditional calculation, Cohen’s *d* = .82. Although the analysis of our own data suggested that the apparent SSM completely disappeared when we applied our unbiased method of evaluating the SSM effect, it is unclear whether a larger effect size based on the traditional method would be completely attributable to the bias in the calculation.

Second, and perhaps more importantly, because the alternative calculation that Adamo et al. (2019) proposed to eliminate the bias requires a specific experiment design that was not followed for our two in-house data sets, we could not include their alternative, matched-layout calculation in the above comparisons. Applying our approach to the data from Adamo and colleagues allowed us to directly compare whether their technique and ours produce similar patterns of results.

To provide some general context with regard to the Adamo et al. (2019) data set: In their experiment, the search target was always a rotated *T* and distractors were offset *L*s, and half were high salience whereas the other half were low salience (controlled via target luminance).^2^ In each trial, there was a single target or two targets, and participants had to click on the targets they found and could terminate the trial by pressing the space bar once they believed they had found all the targets. If the space bar was not pressed within 15 s, the trial timed out. As detailed above, for each dual-target display there were two “matched” single-target displays with the same layout, but with one of the targets from the dual-target display replaced with a distractor.

### Results

Following our recommendations above (see Fig. 6) to evaluate the presence of an SSM effect, we began by comparing the single-target hit rates for the low-salience target to the total hit rate for low-salience targets (regardless of order) in the dual-target trails. Unlike with our data, with their data the single-trial detection rates were significantly higher, *t*(29) = 4.348, *p* < .001, *d* = .79, than the total dual-target detection rates, suggesting that there may in fact be an SSM effect in their data.

**Fig. 9:**
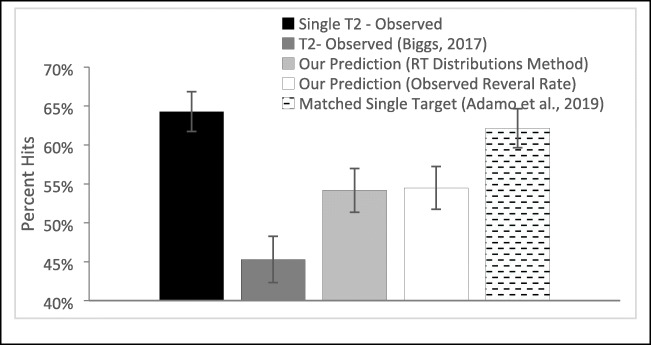
Calculated hit rate for the observed single-target hit rate, dual-target hit rate, as calculated by the traditional (Biggs, 2017) method, and three novel methods—our two and Adamo et al.’s (2019).

To evaluate this possibility, we calculated, for low-salience targets, each subject’s single-target hit rate, the second-target detection rate using the traditional calculation, the prediction of this second-target hit rate using our method that uses the observed switch rate, and the prediction using our method that uses the single-target RT distributions instead (see Fig. 9). Using those four values, we could evaluate the presence of an SSM effect in the same way as we did for our own two data sets. In addition, to evaluate how our method compares with the one proposed by Adamo et al. (2019), we calculated a matched single-target hit rate (see Fig. 9). This is the single-target hit rate based on only those single-target trials whose layouts match dual-target trials that were included in the traditional calculation of second target performance. As mentioned above, Adamo et al. (2019) proposed avoiding bias by comparing second-target hit rate to this “matched” single-target hit rate rather than to overall single-target hit rate. All these various quantities were used to calculate four different SSM measures by comparing the observed second-target detection rate to the four different reference values (see Table 2).

**Table 2:** Test of SSM effect with four different methods

Second-target hit rate (see Formula 2) versus	*t* value	*p*	Cohen’s *d*
Single-target hit rate (Biggs, 2017)	8.953	<.001	1.63
Our prediction (observed switch rate)	5.21	<.001	0.95
Our prediction (switch rate based on RT distributions)	4.316	<.001	0.79
Matched single trial (Adamo et al., 2019)	8.215	<.001	1.5

These comparisons yield a number of key observations. First, and in contrast to the data from our lab, in these data there is a clear SSM effect regardless of method of calculation. This provides evidence that some reported SSM effects are real, instilling some confidence in published SSM effects when effect sizes are large and when the design would minimize the switch rate. Specifically, in this experiment, presenting one target at high and one at low saliency should have reduced the switch rate; the likelihood of finding a low-salience target before a high-salience target should be relatively low.

Aside from indicating that an SSM effect is present, these comparisons yield a second important insight: the estimated magnitude of the effect (measured as Cohen’s *d*) is drastically different depending on method. The methods we suggest result in effect sizes that are about half the magnitude of the effect size for the traditional calculation, whereas the method proposed by Adamo et al. (2019) reduces that traditionally calculated effect size by only 8%. Related, both our expected value calculations for the second-target hit rate are significantly lower than Adamo et al.’s (2019) matched single-target hit rate, both *t*s(29) > 7.7, *p*s < .001, *d*s > 1.4, even though all three quantities are intended as unbiased comparison values for the traditional second-target hit rate in the calculation of SSM effects. This large difference between methods suggests that the method proposed by Adamo et al. (2019) may not fully eliminate the influence of bias. The next section will address this possibility in more detail.

### Is the matching method appropriate?

The issue with the traditional method as laid out in the beginning of the paper, and as first identified by Adamo et al. (2019), is that this method selectively eliminates easy trials from the second target calculation yet includes all trials in the single-target calculation. To match target difficulty across the dual-target and single-target calculation, Adamo et al. (2019) match the array layouts between trials included in both calculations. This method of matching trial layouts in the dual-target and single-target detection-rate calculations rests on the assumption that layout accounts for all (or at least a great deal) of the variability in target difficulty on a given trial.

**Fig. 10:**
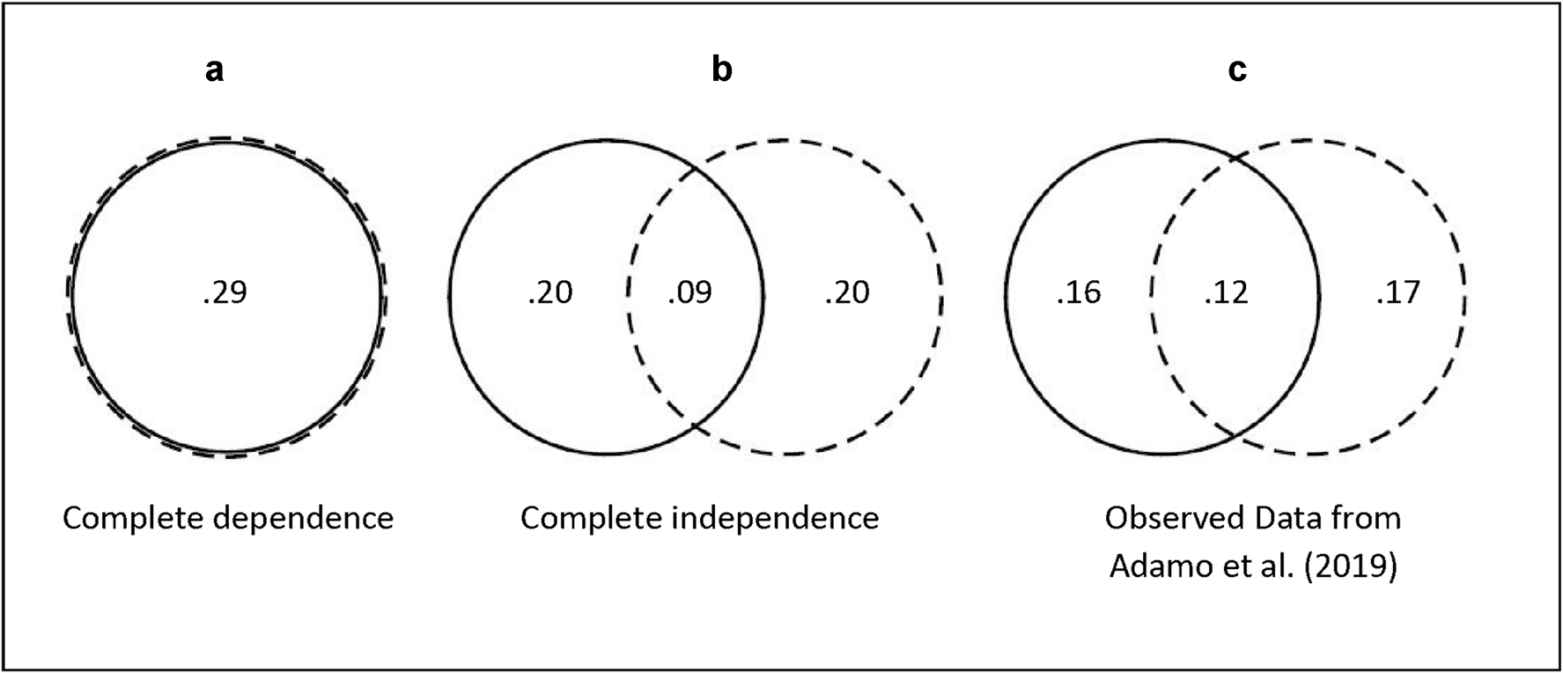
Solid circles delineate the proportion of all layouts that give rise to a reversal in their dual-target trial. Dashed circles delineate the proportion of all layouts that give rise to a reversal across the pair of single-target trials that have that layout. The area of overlap between the circles corresponds to the proportion of layouts that give rise to a reversal by both of those criteria. Panel **a** depicts the prediction under the assumption that holding layout constant equates difficulty across dual and single-target trials (Adamo et al., 2019). Under this assumption the same layouts should be identified by both method so the overlap is complete. Panel **b** depicts the prediction assuming complete independence between the dual-target and single-target criteria (e.g., under the assumption that layout accounts for no variability in difficulty). Under this assumption, only a small portion of layouts should be identified by both methods (the square of the average reversal rate). Panel **c** presents the observed data from Adamo et al. (2019), using dual-target detection order and single-target RTs to identify reversals. In this case, the two methods identify substantially different sets of layouts

To evaluate this assumption, we note that given their method of having triplets of matched layout trials (one with dual targets, and two with single targets), there are two ways to determine trials where the low-salience target is found first—the subset of trials that is eliminated from their calculations. The first method is the one they use; trials with layouts for which the low-salience target was found more quickly than the high-salience target in the dual-target trials are eliminated from both the dual-target and single-target detection-rate calculations. The second method would be to use the reaction times for a pair of single-target trials that are matched in layout, and identify layouts where the single-target RT for the low-salience target was faster than the single-target RT for the corresponding high-salience target. In other words, to use single-target reversals to eliminate trials. The critical thing is that if Adamo and colleagues’ assumption that layout is a good proxy for difficulty is correct, these two methods should identify very similar sets of trials for elimination. By contrast, if the single-target reaction-time criterion and dual-target detection order criterion do not identify the same set of layouts for elimination, it would call into question Adamo and colleagues’ approach. To illustrate a situation of good correspondence between the two methods, we show the perfect case of complete overlap in the trials that are eliminated based on the two methods in Fig. 10a (the proportion of 0.29 in this panel corresponds to the observed reversal rate in Adamo et al., 2019: Their dual-target reversal rate is 28.4% and their single-target reversal rate is 29.0%, which averages to 28.7%).

We also derive a prediction of the overlap in trials that would be eliminated by each method assuming independence of the two methods of calculation. This prediction assumes that the proportion of reversals is the same for dual-target trials and for pairs of single-target trials (an assumption that is reasonable, regardless of the role of layout), but that there is no correlation at all between the layouts identified by the two methods. For this calculation, we again use the proportion of reversals observed in Adamo and colleague’s data of 28.7%. It is important to note that this calculation assumes that layout accounts for none of the variability in target difficulty. Under this independence model, the probability of both methods identifying a given layout for elimination is simply the proportion of reversals squared (the product of two independent processes). This is depicted in Fig. 10b.

Finally, we can use the data from Adamo et al.’s triplets to find how often, in reality, both methods identify the same trials for elimination, as compared with how often the two methods diverge. That is, for each triplet of match-layout trials we identify whether the low-salience target was found faster than the high-salience target in the dual-target trial, and whether this was true for the pair of single-target trials. We then calculate how often these two methods identify the same set of trials for elimination, and how often they do not. These data are depicted in Fig. 10c.

The observed data in Fig. 10c deviate greatly from the perfect concordance prediction that corresponds to Adamo et al.’s assumption that layout explains the bulk of trial-to-trial variability in target difficulty (see Fig. 10a). The observed data are, in fact, more similar to the prediction under the assumption that layout explains no variability at all (see Fig. 10b). Relatedly, out of the layouts that are eliminated by the method of Adamo and colleagues, fewer than half would also be eliminated when using single-trial reversals as the criterion for elimination (0.12 out of 0.28). This result highlights that equating layouts between dual-target trials and single-target trials does not adequately equate difficulty on an individual trial basis. There is, evidently, substantial variability in the difficulty of finding a given target even when layout is held constant.

To summarize, motivated by the realization that SSM calculations involve a selection of dual-target trials that is biased with regard to difficulty, Adamo et al.’s method aims to introduce a corresponding bias in the selection of single-target trials by matching display layout between included dual-target trials and included single-target trials. But because layout is not a good proxy for difficulty, the method does not address the fundamental problem of bias in the calculations of SSM.

## General discussion

Here, we, like Adamo et al. (2019), have argued that the standard method used to evaluate the presence of an SSM effect is biased. It eliminates a number of successful target detections from the dual-target performance calculations, while including all successful target detections in the single-target calculations. This differential inclusion of trials introduces systematic bias in the calculation that could produce spurious or inflated SSM effects. Further, we documented that the magnitude of the bias increases as the order of detections of the two targets becomes more variable; if one target is always found before the other, there is no bias, but the bias increases as the order of detection for two trials becomes less consistent. The magnitude of the bias also scales with the single-target hit rate of the target of interest; the lower the hit rate, the more pronounced the bias.

Having characterized the bias, we then developed a method of using single-target performance data to derive expected values for dual-target performance under the null hypothesis that the detection of one target has no influence on search for the other target. We applied our method to two sets of empirical data from SSM experiments run in our lab. Although the standard method of calculation would have led us to conclude that there was a significant SSM effect in both experiments, these conclusions would have been inappropriate; dual-task performance did not differ significantly from the expected value of performance calculated under the null.

We then applied our method of analysis to a data set from Adamo et al. (2019). That data set had a much larger effect size than our two experiments (as calculated using the traditional method), and although our analyses reduced the effect size by about half, there was still a significant SSM effect, suggesting that some SSM findings persist even when the bias in calculation is removed, albeit with a substantially reduced effect size.

Finally, we used that data to compare our method of calculation with the matching method suggested by Adamo et al. (2019). Our analysis found that our method made a much more sizable adjustment to the estimated SSM effect size, and upon further investigation of their method, we found that it does not address the core problem leading to the bias.

Our findings raise the question of whether existing reports of an SSM effect involved true SSM effects or apparent ones resulting from biased calculations. Unfortunately, most published papers do not report enough data for us to apply the methods we have presented here to those data. Instead, most present only their adjusted dual-task performance (after applying the standard method of dual-task calculation) and report neither the reversal rate nor the full single-target RT data. Thus, the extent to which prior SSM effects are spurious it is still unclear.

Even so, we believe the issues we raise should provoke concern. The data we analyzed here demonstrate that the magnitude of the calculation bias is substantial enough that it *can* produce a spurious effect that is statistically significant, even in a data set of modest size. This fact raises the possibility that other reported significant SSM effects may be spurious. However, here it is important to note that it would be imprudent to conclude based on this report that any specific reported effect is spurious. In fact, when we applied our method to the data from Adamo et al. (2019), we still found a significant SSM effect, albeit with a much reduced effect size. In short, it would be inappropriate to suggests, based on these limited data, that many reported SSM effects are spurious. Instead, we can say with some confidence that the bias in the traditional calculation inflates the effect size in the many reported SSM papers that have used this traditional method of calculation, and may cause some of those effects to disappear.

We also note that a number of existing papers that used the traditional method compared the magnitude of the SSM across different presentation conditions (e.g., Cain et al., 2014) or populations of participants (e.g., Biggs & Mitroff, 2014). It might be tempting to believe that the bias should be constant across these manipulations, and thus conclusions based on differences in the magnitude of the SSM effect between conditions or populations should hold. However, this is only true if the reversal rate and single-trial detection rates are equivalent across populations and/or conditions; a requirement that may not seem to hold in some of these experiments.

In short, without the right subject-level data, we are unable to perform the calculations that would allow us to evaluate the validity of specific previously published findings except those we reported above, and doing so is beyond the scope of this paper. However, we believe that a reanalysis of these data with an unbiased method of analysis is warranted, and we encourage researchers in this area to do so and correct the record where necessary.

It is also worth noting that the standard method we address here is the method that has been used specifically in most visual cognition work investigating the SSM. Most of the radiology research investigating this effect has used ROC analysis (Berbaum, Dorfman, Franken, & Caldwell, 2000). This raises some question of whether the criticism we raise here could generalize to that body of research. Whether the issues we raise affects the radiology literatures depends on whether or not those analyses eliminated successful target detections based on detection order (whether one uses ROC analysis or not is irrelevant). On this point, some reports have included all detections and still found significant SSM effects. For instance, Berbaum et al.’s (1990) influential paper explicitly states that they “included all cases, even those in which the distractor was not detected” (p. 139). However, later in that paragraph they go on to suggest that “a more informative analysis of subgroups of cases may include only cases in which the distractor was detected initially.” In later papers investigating this effect, those authors did rely on analyses that considered only those trials where the distractor was detected first, thereby introducing the potential bias we discuss here. For instance, Berbaum et al. (1994) state, “When all cases in the experiment were analyzed, the satisfaction of search effect was not statistically significant. However, when analysis was limited to cases in which the distracter was reported (85% of the cases), the satisfaction of search effect became statistically significant” (p. 244). This statement demonstrates that, in that case, the effect is only apparent when one eliminates 15% of the trials in which the distractor was missed from the analysis. Given that the likelihood of missing both the distractor and the true target (miss both) is far lower than that of missing only one item (see Fig. 2), the vast majority of these eliminated trials are likely to be trials where the target of interest was successfully detected. Eliminating these detections from the dual-target condition introduces the bias we have been discussing. In sum, although there are clearly papers in the radiology field that have used unbiased methods and found second-target deficits, others have used methods that might introduce bias, and for those a reanalysis of the data is likely appropriate.

### Recommendations for analyses of SSM effects

In future studies of SSM effects, we recommend that, as a first pass, researchers calculate the hit rates for a given target in the single-target and dual-target conditions while including all trials with that target (i.e., without throwing out any of the hits). This is akin to what we did for Fig. 6. If there is a substantial subsequent target cost, the overall accuracy in the dual-target condition should be lower than in the single-target condition, even if it is watered down by also including dual-target trials where the target of interest is found first. If there is no indication whatsoever of such of a reduction, the data have no evidence of a dual-target cost. If there is at least a marginally significant reduction in accuracy in the dual-target condition, there may be a significant SSM effect that is being washed out by the inclusion of trials in which the order of detection is not consistent with an SSM explanation. In those cases, we recommend applying the method we described above, which use the single-target reaction-time data to estimate the expected value of the second-target hit data under the null (see Formula 4, above). This estimate of the expected value should be computed at the subject level, and doing so will allow the use of traditional inferential statistics to compare observed second-target detection rates with expected rates.

As a practical matter, although the simulation method we propose based on single-target reactions times may be ideal^3^ in that it involves only single-target data to generate dual-target predictions, our results show minimal differences between this ideal method and simply using the observed reversal rate from the dual-target trials. So, if it is not possible to generate a predicted reversal rate based on single-target reaction times (e.g., those data were not collected), it is probably acceptable to use the observed dual-target reversal rate. Regardless, doing so would be highly preferable to using the traditional approach.

Finally, while our method can be applied to preexisting data from much of the research, some existing research in radiology did not measure single-target performance for each target. In those experiments, a second “distractor” target was added to scans with native targets (evidence of disease), but there was no attempt to artificially remove the existing native lesions from the scan, thus there is no data from the distractor alone condition. For this reason, neither of our proposed methods for estimating second target performance under the null hypothesis can be used. Here we, again, recommend performing the analysis of total target detections (without eliminating any detections), to see if the dual-target condition exhibits lower accuracy. If it does, one could calculate the SSM effect using the biased traditional calculation, and then compare this calculated SSM effect with the expected level of bias in the calculation for the observed native lesion-detection rate and switch rate. The formula for the expected amount of bias in the traditional calculation that does not require information on the single-target detection rate for the distractor lesion is:

5 $$ \mathrm{F}5\kern0.5em \mathrm{P}\left(\mathrm{A}\right)-\frac{\mathrm{P}\left(\mathrm{A}\right)\times \mathrm{SR}}{\mathrm{P}\left(\mathrm{A}\right)\mathrm{SR}+1-\mathrm{P}\left(\mathrm{A}\right)} $$

In which P(A) would be the single-target hit rate for the native lesion, and SR is proportion of dual-target trials where both targets were detected in which the distractor lesion was detected first. The derivation of this formula is provided in Appendix 1. The magnitude of the SSM effect using the traditional formula and the expected magnitude of the bias for that formula could be calculated at the subject level, and inferential statistics could be used to compare the observed SSM effect to the expected SSM effect due to calculation bias; a real SSM effect would be significantly larger than the expected measurement bias.

## Conclusion

Missing visual search targets can have serious, life or death, consequences in the context of tasks like radiological examination and baggage screening. Thus, attempts to understand the mechanisms responsible for high miss rates may hold the key to developing interventions that target those mechanisms and improve search performance. As such, the work is important and valuable.

However, the high stakes also increase the need for accurate scientific conclusions. What we report here suggests that the traditional method of analysis used in the scientific investigations of the SSM effect is biased and can produce spurious results. Further, recent attempts to address this bias by matching trial layouts (Adamo et al., 2019) fail to fully address the core source of the bias. We emphasize that without the appropriate subject-level data, we cannot say definitively which published papers, if any, may have erroneous findings, but we believe the issues we raise here warrant a reevaluation of existing data. Further, we do not intend to imply that the potential bias in analyses was intentional, but that it resulted from an assumption regarding the method of analysis that may not be justified. Indeed, to their credit, many of the researchers who have investigate the SSM effect continue to try to improve on their analysis methods to ensure that the conclusions drawn are appropriate. We hope that the work we present here motivates those researchers to reevaluate whether their prior results may have been contaminated by the bias we have documented to ensure that the scientific record is accurate.

## Appendix 1

To calculate the bias in the traditional measure, one first calculates the expected hit rate for the traditional calculation. To do so, we begin with Eq. 2 from the manuscript, and then replace the verbal descriptions with the terms used to calculate those probabilities (see Fig. 2). We then reduce the equation to factor the term P(B) out of the denominator. Once this p(B) term is factored out, it cancels with the P(B) term in the numerator, leaving a formula for calculating the expected value for the traditional calculation that does not rely on knowing p(B). In the equation, P(A) is the single-target probability of detecting Target A (the target of interest; in radiology, the native lesion), P(B) is the single-target probability of detecting Target B (the target not of interest; in radiology, the distractor lesion), and SR is the switch rate (defined as the proportion of dual-target trials in which both targets were detected where the order of detection was B then A).

$$ {\displaystyle \begin{array}{c}\frac{\mathrm{trials}\ \mathrm{with}\ \mathrm{B}\ \mathrm{then}\ \mathrm{A}\ \mathrm{detected}}{\mathrm{trials}\ \mathrm{with}\ \mathrm{B}\ \mathrm{then}\ \mathrm{A}\ \mathrm{detected}+\mathrm{trials}\ \mathrm{with}\ \mathrm{only}\ \mathrm{B}\ \mathrm{detected}}\\ {}\frac{\mathrm{P}\left(\mathrm{A}\right)\times \mathrm{P}\left(\mathrm{B}\right)\times \mathrm{SR}}{\mathrm{P}\left(\mathrm{A}\right)\times \mathrm{P}\left(\mathrm{B}\right)\times \mathrm{SR}+\left[\mathrm{P}\left(\mathrm{B}\right)\times \left(1-\mathrm{P}\left(\mathrm{A}\right)\right)\right]}\\ {}\begin{array}{c}\frac{\mathrm{P}\left(\mathrm{A}\right)\times \mathrm{P}\left(\mathrm{B}\right)\times \mathrm{SR}}{\mathrm{P}\left(\mathrm{A}\right)\times \mathrm{P}\left(\mathrm{B}\right)\times \mathrm{SR}+\mathrm{P}\left(\mathrm{B}\right)-\mathrm{P}\left(\mathrm{A}\right)\mathrm{P}\left(\mathrm{B}\right)}\\ {}\frac{\mathrm{P}\left(\mathrm{A}\right)\times \mathrm{P}\left(\mathrm{B}\right)\times \mathrm{SR}}{\mathrm{P}\left(\mathrm{B}\right)\left[\mathrm{P}\left(\mathrm{A}\right)\times \mathrm{SR}+1-\mathrm{P}\left(\mathrm{A}\right)\right]}\\ {}\frac{\mathrm{P}\left(\mathrm{A}\right)\times \mathrm{SR}}{\mathrm{P}\left(\mathrm{A}\right)\mathrm{SR}+1-\mathrm{P}\left(\mathrm{A}\right)}\end{array}\end{array}} $$

To find the bias in this traditional measure, its expected value (calculated above) is subtracted from the single-target detection rate for Target A, P(A). So, the measure of bias becomes:

$$ \mathrm{P}\left(\mathrm{A}\right)-\frac{\mathrm{P}\left(\mathrm{A}\right)\times \mathrm{SR}}{\mathrm{P}\left(\mathrm{A}\right)\mathrm{SR}+1-\mathrm{P}\left(\mathrm{A}\right)} $$

## Footnotes

1 The traditional calculation is unaffected by detection of Target B in single-target trials (see Appendix A), and thus that was not considered as a factor in Fig. 3.

2 Adamo et al. (2019) had a second experiment in which all stimuli were presented at the same saliency, but this rendered the assignment of Target 1 versus Target 2 somewhat arbitrary, so we limited our analysis to their different saliency data.

3 While the reaction-time method of predicting reversal rates has the benefit of allowing an estimate of dual-target performance exclusively from single-target trials, it is worth noting that the reaction time includes differences, not just in search time, but also in decision-making and response selection, which may introduce noise into the predictions made based on reaction time. This noise factor may become particularly pronounced in studies that require the participant to localize the target during the response, in which case the reaction time will also include the time to make the localization.
